# Applying representational state transfer (REST) architecture to archetype-based electronic health record systems

**DOI:** 10.1186/1472-6947-13-57

**Published:** 2013-05-09

**Authors:** Erik Sundvall, Mikael Nyström, Daniel Karlsson, Martin Eneling, Rong Chen, Håkan Örman

**Affiliations:** 1 Department of Biomedical Engineering, Linköping University, Linköping 581 85, Sweden; 2 Cambio Healthcare Systems, Brigadgatan 14, Linköping 587 58, Sweden

## Abstract

**Background:**

The openEHR project and the closely related ISO 13606 standard have defined structures supporting the content of Electronic Health Records (EHRs). However, there is not yet any finalized openEHR specification of a service interface to aid application developers in creating, accessing, and storing the EHR content.

The aim of this paper is to explore how the Representational State Transfer (REST) architectural style can be used as a basis for a platform-independent, HTTP-based openEHR service interface. Associated benefits and tradeoffs of such a design are also explored.

**Results:**

The main contribution is the formalization of the openEHR storage, retrieval, and version-handling semantics and related services into an implementable HTTP-based service interface. The modular design makes it possible to prototype, test, replicate, distribute, cache, and load-balance the system using ordinary web technology. Other contributions are approaches to query and retrieval of the EHR content that takes caching, logging, and distribution into account. Triggering on EHR change events is also explored.

A final contribution is an open source openEHR implementation using the above-mentioned approaches to create LiU EEE, an educational EHR environment intended to help newcomers and developers experiment with and learn about the archetype-based EHR approach and enable rapid prototyping.

**Conclusions:**

Using REST addressed many architectural concerns in a successful way, but an additional messaging component was needed to address some architectural aspects. Many of our approaches are likely of value to other archetype-based EHR implementations and may contribute to associated service model specifications.

## Introduction

**Figure 1:**
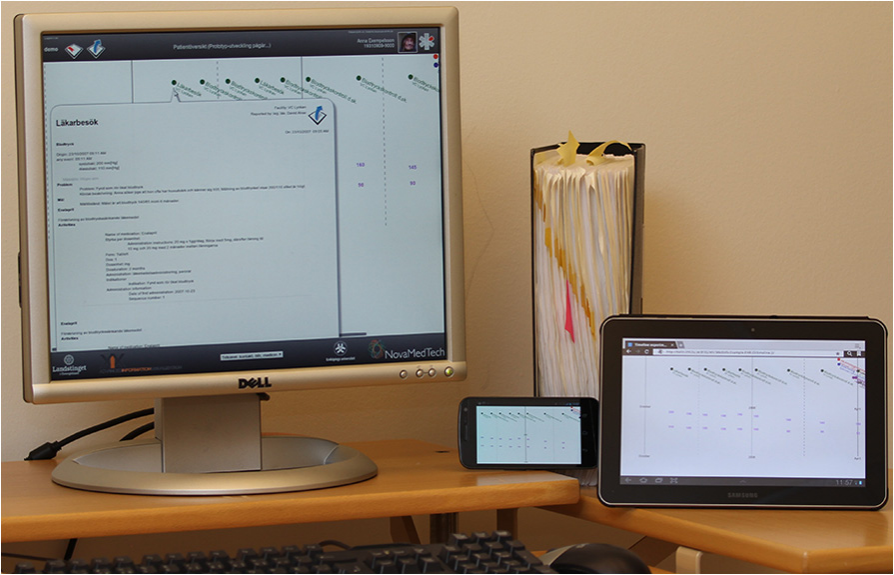
**Prototype platform examples.** The described architecture aims to reduce the time needed to implement prototypes based on openEHR and other archetype-based systems. It has not been obvious for newcomers how to go from comprehensive specifications (the binder) and archetypes to working prototypes (as on the devices above).

There are several intertwined motivations behind this work that together form the requirement context and resulting design philosophy. They stem from years of using, teaching [1], researching, and implementing systems [2-4] based on openEHR and archetypes. Student project participants, clinicians, and software developers approaching parts of openEHR often need a lot of time and effort to get started (Figure 1).

The above-mentioned motivations lead to requirements for an architecture intended to clearly separate concerns, and that should be open, modifiable, scalable, and at the same time suitable for rapid prototyping.

One barrier to understanding openEHR is its size; the complete openEHR specification documents are comprehensive, see Figure 1. The size is justified by the fact that the approach tries to cover the semantic underpinning for systems ranging up to and beyond nation-wide EHR networks for lifelong records [5]. A functional, transparent, and manageable EHR system on which to perform teaching and research in projects related to openEHR and ISO 13606 was needed when the design and implementation of this architecture started. The result had to be straightforward enough to allow new people to get started with relevant parts of the system in short time, for example in Master’s thesis projects or studies aiming for a single scientific paper. A modular design that considers ‘separation of concerns’ by using ‘pluggable’ components of limited functional scope, in combination with examples with reasonable learning curves (using for example HTML and JavaScript) may help newcomers become productive fast.

The approach of dividing the openEHR architecture into functional subcomponents that ease learning and enable decoupled implementation also has potential deployment and scalability implications for full scale EHR systems that require consideration of:

• Openly specified flexible deployment scenarios with partial implementations and solutions from mixed, diversified developers and suppliers backed by different technologies (any HTTP-capable platform/programming language, etc.) Thus facilitating ‘mix and match’ of user interface, decision support, storage, and query solutions, etc., between openEHR implementations.

• How to apply scalability solutions, including the ones built into Representational State Transfer (REST) and HTTP, to openEHR in ‘scale-out’ scenarios, where partitioning the system cleverly and adding more computers scales up the load capacity.

Another design goal was to create an architecture that allows and encourages rapid prototyping and end user innovation, regarding for example user interfaces (including EHR navigation and graphical overviews) and decision support, based on realistic patient data and archetype-based data structures. A goal is to take designs and design stakeholders beyond nice but untested mockups that at implementation time may show to be incapable of coping with real data, since the required source data is not structured and well defined. Many clinicians have well-justified wish lists regarding what an optimal EHR system should do. Prototyping processes based on archetype-based frameworks, should preferably pose the necessary counter-questions regarding how source data can be structured. Structuring has a price as it requires effort both at design time and later at data entry, and that price should be put in proportion to possible gains. A goal is to have these price versus performance discussions early, for example during cooperative prototyping (described by Bødker et al. [6]) and thus help in creating a more complete basis for design than what is often produced in paper-only requirement specifications. Ideally, the proposed architecture should make it possible and efficient to perform many experiments in order to support interaction design, prototyping, etc., processes that usually are iterative and agile.

Iterative experimental prototyping and development of interfaces and services using the proposed REST-based architecture can now (at publication time) be done using LiU EEE (Linköping University Educational EHR Environment). **Appendix A,** “LiU EEE – Implementation details”, describes implementation details of LiU EEE.

LiU EEE is currently used for research and educational purposes. Server side features are mainly written in Java and an XML database [7] is used for storage. Client side features were created mainly in HTML5 and JavaScript. The software is available as open source under an Apache 2 license.

## Background

This background section describes components and the general architectural approaches reused in this work Including Representational State Transfer (REST), URIs, and HTTP.

**Appendix B**, “Introduction to openEHR”, is provided as recommended reading for readers unfamiliar with details of archetype based systems such as openEHR and ISO 13606. It describes openEHR features of importance to the proposed REST-based architecture that need to be understood before continued reading. This includes:

• Design layers: Reference Model (RM), Archetypes and Templates [5].

• Versioning mechanisms and the central objects CONTRIBUTION, VERSIONED_OBJECT and VERSION [8].

• Paths, queries and the Archetype Query Language (AQL) [5,9,10].

The design this paper describes treats EHR infrastructure as a distributed mission-critical system, but the paper does not discuss to what degree EHR systems have (yet) become indispensable, or how healthcare is affected when EHR systems become unresponsive. Thus the background section ends by describing approaches to three desired properties of such systems: scalability, performance, and high availability.

### Representational State Transfer (REST), URIs, and HTTP

Roy Fielding [11] described constraints and design decisions behind the World Wide Web as an architectural style pattern named Representational State Transfer, or REST for short, that was applied to the design of the Hypertext Transfer protocol (HTTP) [12] and Uniform Resource identifiers (URI) [13].

The REST approach aims to support distributed hypermedia systems that scale to Internet size, with general interfaces and intermediary components that reduce interaction latency [11].

Some of the fundamental parts of REST when applied to HTTP and URIs are:

• **Resources** are targets of references and are identified by **resource identifiers** in the form of **URI**s

• Resources can have different **representations** (formats), e.g. HTML, XML, JSON (JavaScript Object Notation), plain text, serialized Java objects. The representations are sent in the **body** of the message. In the HTTP protocol **representation metadata** sent in the **HTTP header** contains things like media (MIME) type. HTTP header fields can also contain cache directives, as exemplified in the ‘performance’ section below.

• A predefined set of **methods** (corresponding to verbs) that act on resources. The method is supplied in the client’s **request**. Methods like OPTIONS and GET should not lead to changes at the server side, so results from those methods can usually be cached. The **GET** method is used to retrieve a representation of the resource that the URI identifies, which is how most normal web pages are fetched. The **PUT** method replaces content at the target URI (or creates it if missing), and **DELETE** deletes resources. The **POST** method is often used to modify or add information, or to perform other operations determined by the server.

• A **response** contains headers, a HTTP Status Code and possibly a body. **Status codes** indicate things like success (including ‘200 OK’, ‘201 Created’, etc.), redirection (including ‘301 Moved Permanently’, ‘303 See Other’, ‘304 Not Modified’), errors in the client request (including the familiar ‘404 Not found’ and the ‘412 Precondition Failed’ that can be returned when conflicts block conditional updates), and server errors (including ‘500 Internal Server Error’). Responses indicating redirection or creation of new resources should contain a ‘Location’ header field with the target URI.

**Figure 2:**
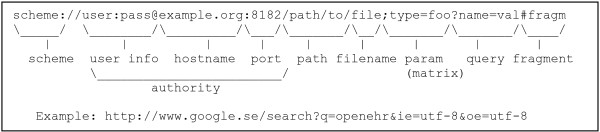
**URI Components.** The components of a URI using the http identifier scheme.

A **Uniform Resource Identifier**[13] identifies an abstract or physical resource. The scheme (for example http, ftp, mailto, urn) indicates how the rest of the URI is to be interpreted (Figure 2).

After the optional user info part, the http scheme, illustrated in Figure 2, specifies which server and port to connect to. The rest of the http scheme URI could be pointing to a specific file in a directory specified by the path, but it could also just serve as an identifier sent to the server that it can process in other ways, for example map to database entries.

The fragment part (after ‘#’) is intended for the client, not the server and if just the fragment is changed, no new call needs to be made to the server. Client-side code (e.g. JavaScript) sometimes uses the fragment to keep track of internal state and to make bookmarking and the browser back button act as expected (by reversing to previous views even when the previous view was locally generated and not fetched from the server).

**URI templates**[14] can be used to specify URI structures with variable parts enclosed within curly brackets, as in http://ehr.imt.liu.se/ehr:{ehr_id}/{object_id}@{versionLookup}

### Solutions and design patterns complementing REST

The REST design pattern over HTTP is not optimized for server-initiated interaction or frequent update notifications [15], which may also be needed in an EHR system. Thus additional design patterns and solutions are helpful, but the difference in scalability properties needs to be considered when selecting where and how to apply them.

#### Event-driven messaging systems

The implementation section about trigger handlers describes how message-processing systems (message brokers) are used in the architecture to report EHR events (e.g. content updates) to interested subsystems like decision support systems and systems copying or sending the newly added data. Message brokers typically have queues or topics that can be published and subscribed to using paths. Our approach aims to keep messaging simple and trigger-oriented; if needed, subscribers can interact and fetch further details via HTTP after being notified of events. Many message brokers include wildcards and regular expressions for selection of topic path and queue path so that subscribers only get notified of things they are interested in. Many brokers also offer filtering based on header content in addition to topic path. Brokers can also handle conversion between messaging protocols and take care of authentication and authorization. In addition to programming language APIs, some brokers can also interact via RSS, instant messaging protocols, and partly via HTTP.

#### Sockets

For small, frequent notifications between components, or between client and server, the POST and GET requests have unneeded features that instead become overhead. A more efficient approach is WebSockets [15] that uses the upgrade mechanism of HTTP to open a bidirectional communication channel over a single TCP socket.

### Scalability - the *ability* to *scale*

Two commonly used scalability approaches are often referred to as vertical and horizontal [16] (chapter 9):

• **Vertical, scale ‘up’** or ‘get bigger’ means getting bigger (often expensive) computers with more processing power and storage when demand increases. For example, this approach can be justified by a lack of time to rewrite existing code to work for horizontal scaling.

• **Horizontal, scale ‘out’** or ‘get more’ means getting more fairly ordinary servers that are not necessarily top of the line, but rather selected for good price/performance ratio. Horizontal scaling is often needed to keep costs down, but requires that the application is designed to run in a distributed environment. Horizontal scaling can involve **sharding** or **MapReduce** approaches and needs to consider the **CAP-theorem**, see below.

#### Sharding

Sharding databases means splitting a database into different partitions that are usually put onto different servers. The effect is that one big database becomes several smaller ones; thus, performance can be increased since the index used for lookups gets smaller and the number of requests to each server is reduced [17,18].

Efficient sharding requires a fast way for processes to figure out which shard (and thus server) to send the request to (see discussion section for details).

#### The CAP theorem

Brewer’s CAP theorem, detailed by Gilbert and Lynch [19] and further illustrated by Browne [20], states the following three desirable properties can not be achieved at the same time in a distributed system:

• **Consistency**, or rather having ‘atomic’ transactions across all involved nodes so that a series of actions are either performed logically as one single operation or not at all

• **Availability**, meaning that the system is responsive so that every request gets a response indicating failure or success

• **Partition tolerance,** meaning that the system keeps working even if some network messages disappear, for example due to nodes crashing or being temporarily cut off from the network

According to Gilbert et al. [19], you can only guarantee two of the above at the same time.

#### Distributed database systems and MapReduce

An alternative to handling distribution at the application level via sharding is to let some storage infrastructure, for example a distributed database, automate the distribution so that the complexities of distributed transactions, replication, etc. are hidden to the calling application. The limitations of the CAP theorem are still valid, but automated solutions often offer tuning alternatives to prioritize between C, A, and P. Since some databases use ‘snapshot isolation’ and ‘multiversion concurrency control’ (MVCC) [21] to reduce the number of potentially performance-degrading locks, it is worth noting that the openEHR time-stamped ‘append only’ versioning system provides a snapshot functionality via the VERSION objects.

Many distributed database systems are capable of running MapReduce [22] tasks that are suitable for batch processing of massive amounts of data in distributed environments by splitting up big tasks into smaller chunks that can be processed in parallel by many computers. For EHRs this is likely useful for example in epidemiology research.

### Performance, caching, and reducing number of requests

Fielding states: ‘An interesting observation about network-based applications is that the best application performance is obtained by not using the network’ [11] (section 2.3.1.3). Designs can strive to minimize the number of calls for example by making a few larger requests rather than many small. Also, by caching (temporarily storing) already fetched data many calls can be avoided. The cached resources could be stored in memory (fast) and on disk (more space available). The local cache of a web browser is familiar to many users, and there can also be proxy servers in the network caching the request on the way to the client. On the server side, repeatedly requested data can be cached in order to reduce calls to disk storage (e.g. databases) and to reuse already performed server-side data processing and compilation.

The headers of the HTTP/1.1 protocol [12] provide many ways to communicate cache-related information that HTTP servers and clients should obey. Some examples are:

• The ‘**expires**’ header (using an absolute date) and ‘**cache-control**’ headers like ‘**max-age**’ (using ranges in seconds) can be used to indicate when a cached response should be refreshed.

• The headers ‘**ETag**’ and ‘**last-modified**’ indicate if or when the content of a resource has actually been changed. They can be used to compare already fetched data to what is currently on the server, and depending on that information, direct how a request should be handled. The ‘ETag’ header contains a string that should be produced by the server in a way that it is changed whenever the content of the resource at that URI changes. The ETag string needs only to be unique related to *different versions* of *the same* resource, but there is no problem if several resources use the same ETag. The ‘last-modified’ header has a resolution of whole seconds and may thus not show differences if a resource has been updated more than once during a second.

• The header ‘**If-None-Match**’, sent from the client combined with the ETag value of a previously cached response, can be used to make conditional GET requests. The server will only serve the page if the fields don’t match. If they match the server sends the HTTP status code ‘304 not modified’.

• In a similar way, a conditional PUT can be performed if the client sends the header ‘**If-Match**’ for example to check that a target resource it wants to update has not already been updated by somebody else.

In a distributed system, also fast in-memory cache data can be shared among networked servers. One approach is to use caching systems compatible with the Memcached protocol [23]. To the programs calling the cache, it looks like a big key-value store (hashtable) that is shared among computers, with the size equal to the sum of available cache memory in all the attached computers. Typically, such a cache is used to store small, frequently used data elements cleverly so that they do not have to be fetched from disk or database as often.

### High availability

Striving for high availability means keeping the system up and running constantly by avoiding single points of failure. Distributed databases and storage systems often have built in replication options to prevent data loss in case of server breakdowns. If sharding is done at application level, similar approaches could be considered.

## Implementation

This section details a suggested way to componentize openEHR-based systems – mainly HTTP-based and programming-language-independent. The technical interface design suggestions can be used as input to future openEHR service specifications. This can be done in the form of an openEHR REST Implementation Technology Specification (ITS).

### Componentization and separation of concerns

The division of components was partly guided by the VERSIONED_OBJECT and CONTRIBUTION lifecycles detailed in the openEHR specification [8]. It was also guided by what was considered to be suitable subsystems with separation of concerns for developers with different interests. In a real production system, these components are possibly sourced from different groups or vendors specialized in for example:

• User interfaces and interaction

• Decision support and other services triggered by added EHR content

• Data validation and conversion based on archetypes, etc.

• Storage and querying (achieving distribution and performance)

**Figure 3:**
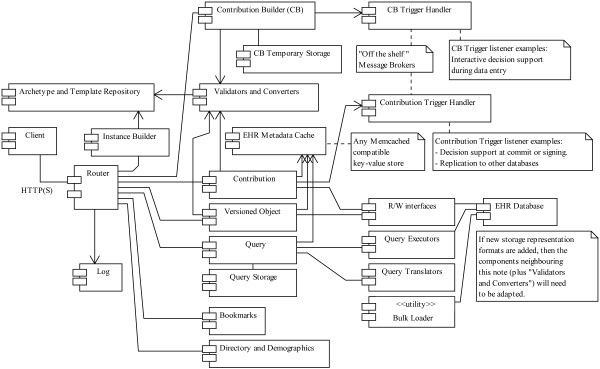
**REST-based componentization of openEHR.** The main components of the suggested REST-based approach and the LiU EEE implementation. The design is flexible regarding component deployment. In a small or experimental setting, all server components can run in the same Java virtual machine on a single computer, and in a full deployment components (and several instances of the same component) could run on separate servers and be provided by different vendors using different technologies.

Some components obviously overlap categories. In the proposed REST approach, many foundational openEHR RM objects are available as separate resources identified by URIs (e.g. objects of type CONTRIBUTION and VERSIONED_OBJECT). Figure 3 shows components and component relationships.

The component communication interfaces are mostly based on HTTP, with the addition of some event-driven messaging via service brokers and caching via the Memcached protocol. Thus even components using a mix of environments and programming languages should be straightforward to integrate. If there is capacity on a single computer to run several components, some external network calls can be avoided by using:

• in-process or inter-process communication if supported by the components or

• using HTTP and other protocols via localhost loopback networking at operating system level or

• using network hardware loopback support (for example when using virtualization on the same machine for components requiring different operating systems)

### Storage considerations and grouping of use cases

In the backend storage, resources (VERSIONED_OBJECTs, etc.) are stored in some *representation* format and potentially decomposed and indexed at some granularity level depending on foreseen major use cases.

#### Conversions and storage format changes

Converters enable HTTP responses in different representations (e.g. XML, JSON or serialized Java objects) to be served from the same stored resource, after media type negotiation based on client capabilities and preferences. This allows a change of backend representation format at later date without changing client interface. However, to avoid excessive conversions, a likely approach is to store representations in the format most often requested by clients in the deployed system.

#### Individual focus versus population focus

Occasionally, discussions about the design of a data storage that balances the need for rapid EHR access in **clinical work focused on individual patients** with performance for **population-wide queries** appear on the openEHR wiki [24] and the openEHR technical mailing lists [25].

In clinical work focused on individual patients, the EHR system often either accesses data as big chunks – documents – in a certain serialization format (for example openEHR COMPOSITIONs in XML format) or as result lists from queries. In this case, it can be reasonable to store data in chunks (e.g. documents) and if possible even in the most frequently requested serialization format and also to index on fields relevant to frequent retrieval needs. Here, this single EHR access usage is called the **single record** use case.

On the other hand, research activities require population-wide queries that access and aggregate particular data fields from large amounts of EHRs. Then it is reasonable to store these as such small pieces in for example a normalized relational database management system or to use some data warehouse approach. Here, this aggregate EHR access usage is called the **multi record** use case and the path component of the corresponding URI is prefixed with /multi/.

Database solutions that have acceptable retrieval speed for the single record use case are not necessarily appropriate for the multi record use case [7].

The proposed REST design allows keeping these two use case families separate in order to provide maximal freedom for implementers to explore diversified approaches. The openEHR append-only (or ‘never physically delete’) principle, combined with its clearly time-stamped operations, simplifies replication from ‘single’ to ‘multi’ databases. If the multi record use case database is not used for entering data, and if a bit of lag-time behind live single record use case EHRs can be accepted, then implementation is rather straightforward.

Neither the suggested REST approach nor the single versus multi division dictates the use of sharding or double databases. Some storage solutions, for example distributed database frameworks and map-reduce-based approaches [22], may prove to be able to handle both the single and multi use cases with good performance and thus may not need the use case separation for performance optimization reasons. However, even with a unified database for the single and multi use cases, there may still be reasons to have the use cases separated at URI level:

• It makes it easier to prioritize computing resources for direct patient care queries higher than statistics and research queries

• Implementation of security can be simplified and optimized if you know beforehand that the query is only allowed to access a particular patient record. Then checking of access control lists for that particular record combined with pre- or post-processing of queries can be done without the need for more advanced query or result evaluation mechanisms.

• Permission to formulate database queries in the multi use case only needs to be granted to users with ethical approval for research, etc. Also anonymization of results may be more applicable to multi than single use cases.

• Patient-specific access logging is given by simply reading the HTTP log in the single use case. For the multi use case query- or result-analysis is required to see if a specific record has been queried.

### URIs to central resources representing EHR RM objects

When accessing a patient’s EHR, a common first view is a list of patient encounters, a problem list, or other summaries; then the clinician fetches details for items of interest. Patient summaries and graphical overviews can for example be based on AQL queries that are used to populate HTML pages with data and hyperlinks pointing to more detailed information. Access to details can be achieved by pointing directly to specific versions in versioned objects using the URI template path /ehr:{ehr_id}/{object_id}::{creating_system_id}::{version_tree_id}.

As exemplified above, central object classes and associated methods from the openEHR specification have been assigned URI patterns for direct access instead of via AQL queries. They are exemplified in Table 1.

**Table 1:** URI patterns for central resources

**URI path template**	
*Example path(s)*	
	Description
**/ehr:{ehr_id}/{object_id}::{creating_system_id}::{version_tree_id}**	
/*ehr:12344321/56d03821-8e89-cca769b7d39e::test2.eee.mi.imt.liu.se::1*	
	Fetches a VERSION, identified by creating_system_id combined with version_tree_id, from the VERSIONED_OBJECT identified by object_id. URIs containing fragments (after a # sign) will have the same effect. Thus /*ehr:12344321/56d03821-8e89-cca769b7d39e::test2.eee.mi.imt.liu.se::1#path* will also fetch the entire VERSION from the server and let the client deal with the fragment.
**/ehr:{ehr_id}/{object_id}::{creating_system_id}::{version_tree_id}/…**	
/*ehr:12344321/56d03821-8e89-cca769b7d39e::test2.eee.mi.imt.liu.se::1/content[openEHR-EHR-SECTION.vital_signs.v1]/items[openEHR-EHR-OBSERVATION.blood_pressure.v1]/data/*	
	Fetches only the part of a VERSION specified by the path after the id. This can be used by applications that for example due to privacy reasons only want to retrieve part of a version.
**/ehr:{ehr_id}/{object_id}/{command}**	
*/ehr:12344321/56d03821-8e89-cca769b7d3/all_version_ids*	
	Fetches object lists or metadata of different kinds depending on command, see openEHR specifications [8] for details.
**/ehr:{ehr_id}/{object_id}@{versionLookup}**	
/*ehr:12344321/56d03821-8e89-cca769b7d39*@latest_version	
/*ehr:12344321/56d03821-8e89-cca769b7d39*@2005-08-02T04:30:00	
	Fetches version based on lookup command, for example the version that was current at a specific time.
**/multi/ehr:{ehr_id}/{object_id}…**	
	Ad-hoc queries in the ‘multi’ use case can also produce lists or reports, sometimes containing detail hyperlinks pointing to versioned objects. By prepending multi to the path such objects can optionally be fetched from, and thus logged by, the multi database instead of the single database, if desired.
**/ehr:{ehr_id}/contributions/**	
*/ehr:12344321/contributions/?start = 1&end = 5&descending = true*	
	HTTP GET returns all contributions for the EHR identified by ehr_id, paged by variables start (default 1) and end (default 20) the default ordering is descending (most recent contribution listed first).
	HTTP POST is used to commit a contribution; all new and changed VERSIONs should be included in the body of the POST. The current LiU EEE implementation accepts either XML or serialized Java objects. Some web-based applications will instead of POSTing to this URI prefer to use the /cb/{committer_id}/{ehr_id}/{cb- id}/commit/ command (described in Table 2) that calls the same verification and storage mechanisms internally.
**/ehr:{ehr_id}/contributions/{contribution_id}/**	
*/ehr:12344321/contributions/7a11c126-e1af-4022-9c36-f046693bb237/*	
	Fetches the contribution identified by contribution_id

Table 1 shows a subset of URI template patterns identifying central openEHR objects. A background to versioning and commands can be found in the openEHR Common Information Model specification [8]. The prefix ‘ehr:’ uses the colon sign so that interface designers more easily can make calls to EHR URIs as described in Section 11.3 of the openEHR Architecture Overview [5]. By prepending a slash (/) to an EHR URI found within data, it will become a fully functional HTTP link to the desired EHR object.

Since the provided LiU EEE implementation is hypertext-driven, these detailed URIs are embedded in the interface pages (like the views in Figure 1) and do not need to be known by end users such as clinicians.

### User interface

The suggested REST architecture does not prescribe clients based on web browsers or any other specific user interface technology as long as it can handle the HTTP protocol and at least the one of the possibly available representations (e.g. XML, JSON, or serialized Java objects) properly; thus Adobe Flash, .NET, or Java (including thick clients) are possible examples.

### Querying

The approach for new queries is to first translate the *clinically targeted* AQL-queries to other *native storage targeted* query languages (such as SQL, SPARQL, or XQuery [26]) and then run the query natively in the database. In the process, a unique SHA-1 checksum of the query, any static query parameters, and the query in its original form are also stored.

AQL is targeted specifically towards archetype-constrained systems based on domain-specific reference models [10]. Thus it targets the application domain and structure rather than the underlying technical storage format, and the AQL queries should be unaffected by differences and changes in underlying solutions that may vary depending on preferences and use case (like the single and multi use cases described above).

#### Query storage, execution, and HTTP redirection flow

The query storage provides permanent facilities for auditing queries sent via HTTP POST, that otherwise would not be stored and logged. This has positive side effects that are described in the results section of this paper.

For the ‘single’ use case queries are POSTed to a URI matching the template: /ehr:{ehr_id}/q/{query-language}/ for example /ehr:12344321/q/AQL/ If the query syntax is invalid, a HTTP error ‘400 Bad Request’ is returned and no query is stored. If the query translation and storage instead succeeds, a HTTP ’303 See Other’ response redirects to a URI according to the pattern:

/ehr:{ehr_id}/q/{query-language}/{query-SHA}/HTTP-compliant clients will then automatically send a GET request to that URI and the response of the query will be returned. When web browsers automate this redirection, the new URL may not be accessible to JavaScript-based user interface code. Thus it is recommend that server implementations also add the URL containing the {query-SHA} pattern in the more script-accessible ‘Content-Location’ HTTP header field.

Most databases support stored queries called with variable parameters, and AQL also supports variable parameters in queries. For performance reasons, any query supposed to be reused should use parameters for variable parts like ehr_id, date ranges, etc., so that optimizations can be performed.

Any parameters starting with ‘_’ (underscore) that are POSTed with the original query are interpreted as being dynamic and are – after removing the underscore – converted to URI-encoded parameters and included in the redirection URI (together with other possibly pre-existing URI query parameters). If the ‘debug’ parameter is present and set to ‘true’, the translated query will be returned as text to the client instead of being executed. Thus the redirected GET request logged in the standard HTTP log contains the unique query-SHA and the dynamic parameter values used in the call. The ‘query’ field containing the original query, additional POSTed static parameters (and metadata about who created the query when, etc.) can later be audited and inspected by calling /ehr:{ehr_id}/q/{query-language}/{query-SHA}/info/. Static parameters can be used for example to send extra configuration parameters to query translators. Static parameters are stored together with the query and included (sorted in alphabetical order as a JSON text string) in the calculated SHA-1 checksum. Example usages of both dynamic and static parameters are illustrated in the LiU EEE implementation (Appendix A).

For the ‘multi’ use case new queries are POSTed to URIs like /multi/ehr/q/{query-language}/ and redirected to /multi/ehr/q/{query-language}/{query-SHA}/.

#### Response formats and hybrid queries

Neither the openEHR specifications nor the AQL description [9] specifies a return format. We have implemented, and are thus suggesting one, for XML-formatted query results. The corresponding XML Schema is available in the LiU EEE implementation.

An interesting option for those queries that don’t need to be standardized and reused between systems, is to formulate the clinical parts of the querying in AQL and then translate and embed those parts inside a query formulated in the (possibly more feature rich) native query language of the underlying database used.

This can be useful for example in an implementation storing both EHR data and ontologies as RDF and uses SPARQL natively for queries. Then, EHR-oriented AQL snippets could be embedded into ontology- or reasoning-oriented SPARQL queries. After translation, the combination has been transformed to a pure SPARQL query that can be executed.

AQL wrapped in a native query language was useful in the epidemiological query study [7], where xQuery aggregation functions missing in AQL were used to wrap some AQL queries.

For XQuery-enabled storage solutions, the flexible XQuery ‘return’ clause can be used to return custom formats marked with any desired MIME-type. Some starter samples that use the AQL plus xQuery hybrid approach and can inspire further experiments are described in Appendix A.

If there are several AQL parts in a hybrid query, they can be translated and combined with the rest of the native query before being executed as a single database query. This can be useful if several AQL queries should be executed and related to each other using features of the native database query language (such as xQuery). If the database has good query optimization routines this could be more efficient than it would have been if several separate AQL queries were executed and their results were combined in application code.

### Caching and reducing number of requests

Even though network traffic should be minimized for performance reasons, having a networked EHR system is necessary when many clinicians, patients, and locations are involved. Sharing EHR data involves transfer and synchronization, so networks and shared storage are helpful.

**Caching** in shared **server-side** volatile RAM memory can be implemented using Memcache-compatible caches. The cache tracks when and by which contribution each EHR was last updated. When potentially modifiable data is requested, the RAM cache is inspected to see if there already exists any entry for the requested EHR ID, and if not, the latest EHR modification time and the ID of its associated CONTRIBUTION is fetched from the database and stored in the RAM cache. Whenever data for an EHR is contributed, the cache is actively updated. This server-side caching aims to reduce database requests by feeding the production of HTTP header fields used for HTTP client caching as described below. (In-memory caching needs will likely differ depending on implementation and deployment; thus this exact approach may not fit all.)

**Caching at the HTTP level** is a client + server cooperative behavior. The outlined REST-based architecture does not force clients to be based on web browsers, and it certainly allows ’fat‘ clients, i.e. installed programs or ’apps‘ that have most behavior and GUI locally and only exchange EHR data over the network. There are also automated applications like server-side decision support systems and report generators where a browser-based approach does not make sense. For performance reasons, such non-browser-based programs should still act according to HTTP and cache information from the server just like a web browser does. To make implementation easier, it is suggested to base clients on existing capable HTTP client frameworks that negotiate and handle caching, etc., rather than using raw HTTP connections without such support. The caching currently used is simplistic but reduces unneeded database requests:

• Resources that don’t change are returned with a high (server-side configurable) ‘max-age’ cache HTTP header value, but no ETag since update checking is irrelevant. They can thus be cached by the client and intermediate proxies.

• Static resources, like user interface components, JavaScript files, etc., that do not contain sensitive information may be cached in public proxies shared by many users. They should be marked with the ‘Cache-Control: public’ HTTP header in addition to high ‘max-age’. In a production system, such resources may be served from separate optimized http servers instead, in order to improve response time and reduce load on application servers that interact with databases.

• EHR content resources that don’t change, like particular VERSIONs of VERSIONED objects with URIs like {object_id}::{creating_system_id}::{version_tree_id}, should also be returned with (another separately configurable) high ‘max-age’. Such EHR data should be served with the ‘Cache-Control: private’ header, since requests from separate users should not be intercepted by shared proxies but instead be separately logged at originating servers. In some use cases, such resources could technically be privately cached for infinite time. In practice, however, policies may require that new requests are made each day, or after an average user session length of time, in order to log repeated access on the server side or to check if access permissions have been changed lately.

• Patient-related resources that may change, like AQL query responses or URIs on the form {object_id}@latest_version, should be returned with latest-modification and ETag headers based on the latest contribution for the patient’s entire EHR. Thus if the ETag of client and server match, no database request will be made and a ‘304 Not modified’ response will be returned. Changes to access control rules may also change what should be returned to a client, but since changes in EHR_ACCESS objects are also submitted as contributions they will also cause server-side ETag changes and thus automatically cause revalidation of results.

### Contribution Builder

The Contribution Builder component provides a temporary ‘writing space’ to the users (detailed in Table 2). The space is divided into separate compartments for each EHR the user wants to edit (since they might have several ongoing edits at the same time). It is even possible for a user to have several separate writing spaces (contribution builds) for the same patient at the same time, the first one for each patient will be named ‘default’. The contribution builder URIs in Table 2 are targeted to developers and not intended to be seen by end users.

**Table 2:** URI patterns for the Contribution builder

***URI path template***	
*Example path(s)*	
	Description
**/cb/**	
	Shows a page describing Contribution builder usage and links to further pages
**/cb/{committer_id}/**	
	Lists EHR IDs for which the user has active uncommitted contribution builds
**/cb/{committer_id}/{ehr_id}/**	
*/cb/dr_who/12344321/*	
	Lists active builds for ehr_id by committer_id
**/cb/{committer_id}/{ehr_id}/new-cb-id/**	
	A POST request creates a new empty Contribuition Build and redirects to it. A query parameter 'description' can be used to set a customized name of the contribution build.
**/cb/{committer_id}/{ehr_id}/{cb-id}/**	
	Lists the VERSION objects contained in the selected build and commands to view, modify, or delete those objects or the data contained in the versions. A form that can be used to create new VERSION objects is also provided. (Often the contribution_id will have the value ‘default’)
**/cb/{committer_id}/{ehr_id}/{cb-id}/{temp-id}/**	
	GET shows a page that allows viewing and editing of metadata for the VERSION object identified by temp_id
	DELETE deletes the VERSION object
	POST can be used to modify contents
**/cb/{committer_id}/{ehr_id}/{cb-id}/{temp-id}/data/**	
	GET views the data field of the version identified by temp_id. If the application/xml media type is requested by the client an XML serialization of the data is returned, but if a text/html media type is requested, a html view of the data including a data editing interface is returned.
	PUT replaces the contents of the data field
	POST can be used to modify data contents
	Converters can be implemented and attached to this resource to accept data entry in more formats.
**/cb/{committer_id}/{ehr_id}/{cb-id}/new/{command}/**	
	POST is used to create new VERSION objects with the data field based on different sources determined by the command. Valid commands are:
	• **update-version**: creates a new version based on an object that already exists in the EHR, it will be stored as an updated version of the original when committed.
	• **copy-version**: creates a new object based on a copy of an object that already exists in the EHR, it will be stored in a new VERSIONED_OBJECT and not be related to the original.
	• **from-form**: bases data completely from the form variable named ‘data’
	• **from-url**: the data field will be a copy of the content of a resource identified by the ‘url’ field in the POSTed form with the request. This command is useful for example in debugging and testing.
	• **from-instance-template:** like ‘from-url’ but the file at the url will be interpreted as a FreeMarker template and that will execute in a context populated with all variables sent in the POST request. This command allows a simple way of combining data in ordinary html forms with a skeleton structure provided by an instance example where target values have been replaced by variable names. This makes form field names flexible. (The FreeMarker template engine is only available in Java; thus some other templating language or mechanism for variable substitution should be investigated if this approach is used in an openEHR service specification.)
	• **from-ehr-path**: like ‘from-instance-template’ but the form fields must be named as EHR paths corresponding to nodes that will be replaced in the file identified by the ‘url’ field.
	Note that the last three commands create outgoing GET requests and thus can be **exploited for denial-of-service** attacks by malicious (but logged in) users or pose an **information disclosure risk** if URLs to external sites are allowed. Many HTTP clients include a ‘Referer’ field in the header that in this case may include both committer_id and ehr_id that could be transmitted to the external system if implemented badly. In the LiU EEE implementation, the ‘Referer’ field is instead set to the hostname of the system followed by the path ‘/static/restricted.txt’ where an explaining file is placed.
**/cb/{committer_id}/{ehr_id}/{cb- id}/validate/**	
	GET returns a validation report indicating validity or errors of all VERSIONs in the contribution build
**/cb/{committer_id}/{ehr_id}/{cb- id}/commit/**	
	POST commits the entire contribution build as a CONTRIBUTION and then deletes the build. If the commit is successful, a ‘201 Created’ response will be returned containing the contribution ID of the newly created CONTRIBUTION.

Table 2 shows a subset of URI template patterns identifying the Contribution builder parts.

A ‘fat’ client can have its own internal contribution builder mechanisms and commit an entire contribution to the database via the contribution resource in one step. ‘Fat’ clients can reuse the REST semantics and the components to make client development and maintenance easier. A client-side contribution builder can store data temporarily if the network is unavailable, for example in mobile applications.

A user’s contribution builder URI only needs to be accessible to that user, and when a particular contribution build within the builder is finished, validated, and committed, it is deleted. The only sharing done between clients is when the end user application is configured to share the same active contribution build between different devices that a user is simultaneously working on. Thus the contribution builder does not have the same distribution issues as shared EHR content; instead, the main issue is to be fast and responsive to the end user.

The purposes of the **contribution builder** are pedagogical and technical:

• It aims to lower entry-barrier and simplify development of data entry clients by allowing incremental learning (also via examples) and validation to help detecting misunderstandings and errors early. In addition to assisting end user interface development, the same approach can be used to develop data entry mechanisms for medical devices and external systems.

• It reduces the number of network calls and writes to the shared core databases by collecting all changes to a single contribution submission HTTP call. A result of this is that the core servers do not need to keep track of resources with user-modifiable state between calls; instead, this is done by the non-shared user-specific contribution builder resource.

• It allows shared and clearly specified ways to attach interactive decision support to data entry components, possibly from different developers.

### Validating RM instances using archetypes and templates

Validator components can validate RM instances (EHR data) on different levels. Firstly, RM instances can only be instantiated according to the object model of the RM, meaning conformance to the naming of classes and attributes, correct types and optionality, and cardinalities of attributes. Secondly, RM instances can be validated according to the archetypes that were used to create these instances (archetype constraints can be quite permissive though). Thirdly, when templates are used for data entry and creation, specialized constraints on the template level can be used to further validate RM instances.

The ‘rm-validator’ component in the openEHR Java reference implementation[2] is designed to perform archetype- and template-based validation with a single API. In fact, because of the latest development around ADL and AOM 1.5, there are no longer any significant technical differences between an archetype and a template. This makes it possible to create a single component for archetype- and template-based RM instance validation API.

### Instance builder and RM-skeleton

The resource called ‘instance builder’ can provide valid example RM-instance skeletons that can be used for working with an archetype or template for user data entry purposes. Such example structures can be modified and used as outlined in Table 2 under ‘from-instance-template’ or ‘from-ehr-path’. However, not all GUI generation and data entry approaches will have use for this kind of ‘instance builder’ component.

In the openEHR Java reference implementation, a component named ’RM-skeleton’ was designed to generate example RM structures directly based on constraints in a user-provided set of archetypes or templates. The component traverses a given archetype or template and generates an example RM structure valid to the constraints. Different strategies can be used for RM-skeleton generation. For instance, a minimum strategy will result in the generation of valid RM instance that is according to the minimum requirements of the given archetype or template. If a maximum strategy is chosen, all optional structures are populated on RM level. On the leaf value level, default or assumed values are used when populating instances of data types if they exist. For numeric data types, valid values in the validation range are used.

The RM-skeleton component can be used to create an ‘instance builder’ implementation in Java based systems, as was done in the LiU EEE implementation (described in Appendix A).

### Bookmarks

**Figure 4:**
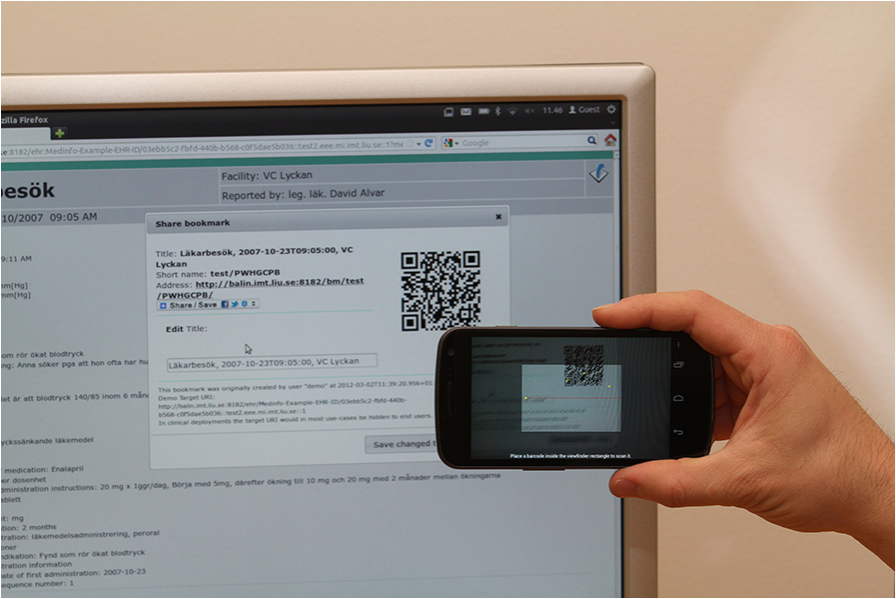
**Bookmark sharing.** Using QR barcodes is one way of sharing EHR bookmark links to camera-equipped devices.

In a collaborative setting, needs to share pointers (references) into the EHR contents may arise, for example to share a particular view of the EHR to participants of a clinical conference, or for a patient with EHR access to ask questions concerning a part of the EHR. Such pointers can also be useful for an individual clinician to share links among different devices such as laptops, tablet computers, smart phones, or projector-connected computers (Figure 4).

All URLs defined by the architecture can be bookmarked and shared via email, instant messaging, etc., just as any other web link. Correctly used, such links can be beneficial for providing pointers to EHR content, perspectives, and focus points between different presentation devices and users. Users familiar with web links will probably grasp the concept of EHR links, their possibilities, and their implications. Recurring use of bookmarks and links may also bring attention to the potential benefits of linking within EHRs using the openEHR and ISO 13606 linking constructs in order to create for example medical history descriptions or discharge summaries without repeating too many details.

#### Sharing and privacy

Examples of EHR focus points and perspectives can be pointers to a specific progress note or a link to a perspective showing all events from certain months in a maternity overview. In the proposed REST architecture, URLs are used to represent pointers. The fragment part of a URL (after ‘#’) shall be interpreted by the client [13], so the URI http://ehr.lio.se/MaternityOverview?ehr=123400&s=1999&e=2001#f=1999-12-20T15:30;w=P2M can define both what to fetch from server and where the client – for example via JavaScript in a browser – should zoom in to and focus in the fetched data.

EHR content retrieved via any kind of URL is already protected by access control by the router component and thus requires authentication and the right permissions to be read. However, full length URLs themselves partly expose EHR structure, especially deep paths into content details or usage of specialized summaries. In some cases, the URL can be used to derive knowledge about a patient even if the receiver of a shared or accidentally leaked link does not have permissions to resolve the URL and retrieve the document content. A service that preserves privacy by producing shortened, opaque replacement links that do not expose EHR structure is thus justified for links intended to be shared outside a controlled environment. By making creation of bookmarks with such opaque links easy and by avoiding exposure of potentially privacy sensitive URIs in the clinical end user interface, the risk of unwanted information leakage can be reduced.

Short URLs are also more convenient with respect to line-breaking email clients, spoken and handwritten messages, limited-length media such as text messages, and barcodes such as QR codes. A versioned object with the URL http://ehr.lio.se/ehr:1234000/2d4b-4e3d-a3f3::ehr.lio.se::2 could for example get the shortened URL http://ehr.lio.se/bm/ep7TtN that could be shared as text or encoded as a QR code and captured by cameras in mobile phones and other devices.

When resolving the bookmark, a 303 ‘see other’ redirect containing the bookmark target URI is sent to the client by the bookmark server. Thus the client automatically makes a new HTTP request to the actual target resulting in execution of normal authentication and logging at the target server.

### Triggers

When updates to an EHR occurs, event listeners can be triggered and perform tasks such as activating decision support. One option is to integrate such triggering in the code driving the user interface, but then it becomes tightly coupled to that specific GUI implementation. A less GUI-coupled option in the suggested REST partitioning is to trigger when EHR updates are made via the contribution and contribution builder resources.

**Contribution** updates activate triggers. In openEHR, contributions can be flagged as complete or incomplete (for example when having to commit unfinished work). New contributions are also made when attesting previously committed data. After a contribution is made, the submitted information becomes visible to all authorized users and parts of the system. Interested contribution trigger listeners can be components for decision support, system internal replication, system external export, etc.

Another mechanism is needed if decision support routines are supposed to interact with users during data entry (when decisions are being made) rather than at commit or attestation (when many decisions have already been made and can only be critiqued). This kind of interaction can be compared with the support given by interactive spell-checkers that underline misspelled words.

If GUI code is designed to update the data representation in the **contribution builder** whenever fields of interest are updated, then contribution builder trigger listeners can interact with users during data entry.

The theoretically interesting parts of the trigger mechanism is the semantics of the messages sent via message brokers; the messages from the ‘Contribution Trigger Handler’ only identify the patient, the committing user, the changed resources, and the archetypes and templates that were involved. Trigger listeners can then fetch more detailed information by using normal HTTP calls to Versioned Object resources, etc.

Technically, messages in the suggested architecture can be sent via the Simple Text Orientated Messaging Protocol (STOMP), see http://stomp.github.com/, and can thus be processed by many different message brokers and converted to messages via JMS, Websockets, XMPP, RSS-feeds, etc. STOMP messages can also be consumed directly by client frameworks available in many languages for example Java, C, C++, C#, Ruby, Perl, Python, PHP, ActionScript/Flash, Erlang and Smalltalk.

## Results

The design decisions regarding how to split openEHR into manageable pieces and also to use REST over HTTP resulted in benefits but also involved tradeoffs, and these results are presented in this section.

### Familiar bookmarking support

By giving each interesting piece of the EHR a unique URI, normal bookmarking and link sharing, familiar to web users, automatically becomes possible. In browser-based interfaces, bookmarking and link sharing became so easy that care had to be taken when deciding what URIs to expose to end users. This prompted the creation of the bookmarking service.

A consequence of bookmark usage is that system owners should insist that new user interfaces are also able to identify their state using URIs, possibly including URI-fragments.

### Client caching included in HTTP

Since the HTTP protocol already included comprehensive cache control and negotiation the only thing needed to leverage this was to populate the correct headers. By providing suitable ETag and max-age cache controlling headers, the rest of the client–server caching behavior was gained ‘for free’.

### User action logging almost for free

The HTTP server logfile, combined with the storage and GET-redirection of POSTed queries, reflects user actions; it includes who did what when and the referring page. The web server log can probably be used unmodified for EHR read log analysis using ordinary tools for web log analysis and statistics.

The REST part of the system uses HTTP for most component interactions. With properly designed URIs that reveal the semantics of the operations, logging and monitoring of HTTP calls provides most of the information needed for audit and system maintenance. The CONTRIBUTION mechanism, which is mandatory for write operations in openEHR, records what has been changed, and the URIs reveal the ID of the committed CONTRIBUTIONs. Thus all things needed to audit EHR writes are implicitly given by existing mechanisms.

When it comes to logging read operations, things like accessing single VERSIONS goes over HTTP and is logged automatically, but information can also be read via queries. For queries sent by HTTP POST, the act of querying is automatically logged, but the POSTed query itself would disappear if it was not separately stored. However, in this system the queries are already stored and redirected to a new URI that also gets logged, so this is not a problem here. Other POSTs of importance, like creation of new EHRs and bookmarks, also redirect to the URIs of the newly created resources, and as long as such created resources are version-controlled (append only), full audit is possible.

To get a complete picture of the system, administrators will – in addition to HTTP server monitoring – have to monitor the actions and logs of cache systems, message brokers (trigger handlers), and databases.

### Representation format conversions and upgrades

Due to the separation of resources and representations in REST, the basic ‘plumbing’ of the system (URIs, etc.) remains intact if new media formats are added in the future. The storage format in the backend can change without changing clients, given that the following are provided:

• media type conversion mechanisms

• AQL query translators targeting the new storage backend (At the time of publication only AQL to XQuery translation has been created.)

### Fast prototyping

The fast prototyping aim of the architecture was recently tested in a project that asked for a graphical patient overview application compatible both with web browsers on regular computers and with touch interfaces on mobile devices. Using the LiU EEE implementation of the architecture and AQL combined with HTML5 and regular JavaScript libraries, acceptable prototypes could be created and modified faster and easier than we had expected ourselves. For example, an interactive graphical patient overview was created with a less than 500 lines of sparse and well commented JavaScript and HTML code, including the three AQL queries it was based on. Admittedly subjective, we still think readers would find this result of interest and encourage further modification and creation of new visualizations.

GUI prototyping efficiency and speed is helped by the fact that no application restart is required in order to see the effect of changes in for example HTML, or JavaScript files. By simply saving the edited file and reload the page in the browser, the edited GUI is immediately visible.

### Benefits from storing and redirecting POSTed queries

The query storage and redirection mechanisms introduced for both auditing and caching reasons have additional benefits:

• The central shared database engine is protected from reparsing equivalent queries re-posted by badly designed GUI code since the comparison of SHA-1 checksums is done before calling the database.

• If a frequently accessed query is manually optimized by database administrators, that particular query URI can easily be redirected to specialized processing or servers using regular URI-based redirection.

• For implementations where there are reasons for not fully optimizing every query, the query storage can be designed to keep track of how often every query is run, and when reaching a threshold a frequent query can be optimized (for example by being converted to a stored procedure or similar approaches).

• A two-level GUI design process is encouraged: at one level develop the query and parameters to get the URI, at another level just include a URI as a GET request in the GUI to fetch data.

• By saving query result files, preferably from artificial test patients, using the same relative URI hierarchy as on the server, many GUI artifacts can be designed and implemented by external designers using a file directory with relative URIs without needing access to any servers. This reduces many potential technical and privacy-related obstacles during design.

### Tradeoffs resulting from the separation of concerns

The componentization of openEHR was intended to separate concerns between components so that different developers or different vendors could focus on different parts. However, functions from some components are hard to separate clearly, leading to potentially confusing dependencies among components:

• The **Contribution** resource should only be concerned with Versioning semantics and storage, and not care about clinical content semantics; thus it calls the **Validators and Converters** to validate clinical content before committing it, but the **Contribution Trigger Handler** may need a list of archetypes used in the commit in order to alert the correct subscribing **Decision support** components. A workaround forces the **Validators and Converters to** make a list of archetypes used and send it along to the Contribution resource that then sends it to the **Contribution Trigger Handler** after commit.

• Another entangled component is the **EHR Metadata Cache** that gets used by many resources. If these resources are distributed to several servers, then distribution of the cache may also need to be carefully considered.

### Benefits resulting from sharding on EHR-ID

In the case of distributed, writable openEHR storage, consistency (C) is needed between each writable copy of a specific patient’s EHR at the instant when new CONTRIBUTIONs are written, in order to assign the correct id to new VERSIONs within the CONTRIBUTION. Thus, if partitioning (P) of a certain EHR can temporarily be avoided by directing all writes for that EHR to a single computer (or a single consistent, non-partitioned cluster of nodes), the system will be able to provide availability (A) in the sense that the returning HTTP response can tell the client if the write succeeded or failed.

### Tradeoffs resulting from sharding on EHR-ID

Sharding – either manually at application level or automated by distributed storage frameworks – can allow scaling out by adding more servers, but it also leads to application complexity if queries and update transactions span over several shards of data.

#### Merging records

It may be necessary to merge data belonging to different EHR-IDs. One example is patients that have not been identified initially and thus have gotten a new EHR-ID assigned, but may later become identified. A second example is data coming from different healthcare providers that unaware of each other both have created EHRs for a patient. A third example is when data for one patient by mistake happens to be entered in some other patient’s EHR.

The openEHR specification [8] covers mechanisms for merging records, but the suggested sharding may have caused the records to be in different shards on different servers. Data from the source EHR should ideally be logically deleted in the same transaction as they get written in at the destination EHR. This can be solved by two-phase commit approaches involving both servers, but such solutions require extra thought and program code to be maintained and can be considered a negative tradeoff consequence.

#### Simultaneous retrieval or change of many EHRs

Atomic queries retrieving data simultaneously from several patients can be desirable not only in multi use cases working with slightly delayed data, but also in direct patient care that needs consistent up-to-date data. Examples include group summaries of all patients in a ward or a listing of all patients booked for a certain procedure. Getting a consistent read-only view at a certain time-point can be achieved by sending the current time as part of the query and then for example:

• wait the for ‘multi’ backend (if you have one) to catch up to that time-point and then run the query, or

• direct the query (including time-stamp, somewhat analogous to MVCC) to all shards and then collect and merge the results (a MapReduce-like behavior).

Group transactions that involve writing are trickier and will need a two-phase commit involving the servers of all patients in the group, as discussed in the merging tradeoff above.

Due to the limitations described by the CAP theorem, it is preferable if atomic cross-shard operations – especially involving writes – in a distributed system are exceptions, permitting waiting times that exceeds the time of temporary network partitions, rather than if they are common in normal, time critical usage.

## Discussion

A REST architecture seems to fit many openEHR use cases. Implementations automatically acquire many benefits from the architecture of the World Wide Web as described above (together with some tradeoffs).

By publishing this architecture and resulting findings we hope to inspire discussion and invention regarding design of archetype-based systems and hope to provide input to an openEHR service specification using REST supplemented with some messaging. This does not exclude the possibility for also creating a parallel openEHR service specification based on for example SOAP-encapsulated remote procedure calls.

### Applicability to other existing or future Archetype-based models

Most parts of the described approach should be applicable also to future updated openEHR versions that may come out of for example the Clinical Information Modeling Initiative (CIMI), http://informatics.mayo.edu/CIMI/. Also other archetype related formalisms such as ISO 13606 and Multi-Level Healthcare Information Modelling (MLHIM), http://www.mlhim.org/, should be able to use our approach in implementations that adopt a similar versioning mechanism. If a different reference model is used, the modules that need change are primarily the Validators, Converters, and Query translators.

### Need for sharding

The described REST slicing does not impose sharding on EHR id; it only makes it easy to shard already at the URI level if you would ever want to.

As long as it is technically and financially possible to scale ‘up’ by getting capable hardware for running a single, sufficiently large, database backend at the same pace as the load increases, or distributed frameworks that automate distribution can be used, sharding at the application level is not necessary.

### Sharding key mechanisms

The approach described in this paper suggests shards that only contain some EHR IDs each, but the sharding mechanism is left open to implementers. Some shard allocation alternatives to explore are:

1. mathematic algorithm using EHR ID as input (for example modulus operations or other hash functions)

2. a directory lookup server, preferably cached, associating each ID with a shard based on some static or dynamic parameter like

a. geographical datacenter closeness to where the patient lives or is treated most often

a. recent or frequent use

a. birth month or day of month (this is sometimes used for paper health records since it gives a fairly constant population division size allocation).

A benefit when using directory lookup sharding mechanisms is that EHRs can easily be moved between shards, for example when maintenance or cluster re-balancing is needed.

### Terminology system binding and querying

Under the broad concept of binding of information models to terminologies, several activities have pointed to the necessity of principled approaches [3,27-30]. Most of the work done so far has studied the problem of terminology binding at design time, i.e. the study of the problem of integrating information and terminology models or the proposal of solutions or demarcations between these two representational alternatives. However, systems working with structured representations in run time have additional requirements. Given terminology-bound information models, the system must support the storing and querying of terminology-bound patient information instances, most probably allowing post-coordinated terminology expressions and possibly even allowing for the bridging of varying terminology binding solutions [31]. Partial implementations addressing these issues are described in Appendix A.

Post-coordinated terminology expressions, as pioneered by the GALEN project [32], will also have to be considered in future implementations. Due to the combinatorial nature of the biomedical domain, possibilities of post-coordinated expressions, allowing for the uniform querying of post- and pre-coordinated terminology expressions is necessary.

### Is a REST architectural style appropriate for EHR deployments?

The REST architectural style was intended for Internet-scale, distributed hypermedia systems, and it can be discussed whether it is appropriate to apply it for an average EHR system, especially considering that such systems might be deployed in a fairly regulated and controlled environment perhaps even within a single organization with limited geographical distribution. Some scaling aspects of REST are likely not necessary for such settings, and the REST constraints might have side effects that could be avoided if some of RESTs constraints were lifted. Web-scale approaches may still be of value in the future deployments if the following considerations are relevant:

• The amount of patient data is increasing due to increased use of data-intensive clinical methods such as monitoring systems, 4D imaging, and ‘omics’ applications. Combined with desires to keep lifelong health records, this calls for thinking about massive scalability early in design.

• The content of a particular patient’s EHR is not served to millions of users, so some scaling approaches of popular, non-personalized web sites are likely of limited use but the change detection capable caching of queries (for summaries, etc.) that may be accessed repeatedly during a patient session can reduce the total number of database requests.

• Design experiences regarding multi-organizational aspects of the web, including trust and security issues across intermediaries, may be of value within a healthcare organization having a system of components from different vendors.

• Experiences from the gradual upgrade and heterogeneous mix of clients and servers used on the web may be applicable within health organizations interacting with many other organizations. Also being able to gradually upgrade organization-internal systems may help in detecting errors and making improvements before launching new versions to the whole organization. It is even possible to gradually upgrade media formats used for serialization and communication (for example going from XML to JSON or some binary format) without having to re-learn architecture or upgrade all parts of the system at once. This is useful if many vendors changing at different speeds are involved in the same deployment.

### Other drivers for using HTTP and web-based clients

The suggested REST approach does not enforce the use of browser-based applications, but certainly fits them well. Reasons to go towards web solutions are many:

• Technology improvements and standardization effects of the web drives costs down, and capability up, for web clients including mobile devices. This contributes to making new devices attractive for healthcare usage too.

• Web-based solutions reduce client maintenance and automatically upgrade when the server does.

• Long-lived infrastructure is of value when aiming for lifelong EHRs, and by using web technology systems may have potential to evolve gracefully as long as the web does.

### Notification of bookmark changes

A bookmark targets a point in the live EHR and does not constitute a static screenshot. EHR content and access restrictions may thus change between the moment of bookmark creation and retrieval. The bookmark can only be resolved by authenticated users with permissions to read at least parts of the original target content. At bookmark resolve time, users should be alerted when there is more or less information available as compared to when the bookmark was created. Table 3 shows warning policy options.

**Table 3:** Bookmark resolution options

	**Block resolution completely**	**Warn user**	**Resolve bookmark without warning**
More info	Irrelevant	Good	Potentially confusing
No change	Irrelevant	Irrelevant	Good
Less info	If required by law or policy	Good	Dangerous

Table 3 illustrates bookmark resolution options. The bottom right box says it can be dangerous to, without warning, show EHR content accessible by the user at bookmark resolve time if there is less information available to this user than when the bookmark was created (possibly by another user and at another time).

Bookmark target change detection needs to be considered in implementations. It is easy to calculate that the target of a bookmark *may* have changed by checking if any EHR content or access rules have changed since the bookmark was created, but this is likely of little value for the end users who will want to know if it has changed for real or not. Comparing the bookmark target versions at creation and retrieval will give the real difference, but since bookmark lookups are expected to be frequent, efficient change detection routines are needed.

### Security and confidentiality considerations

A proper security and confidentiality analysis (out of scope in this paper) would be needed before deploying a system based on this architecture with ‘real’ patient data.

It is fairly easy to track deep detailed URIs when viewing HTML source or monitoring unencrypted network calls. If, on the other hand, HTTPS is used when crossing trust boundaries the only thing seen on the network is that a certain server and client are communicating (the URIs can not be seen). To a high degree, security then involves the same issues facing all networked systems.

### Future work

The exploration of REST applicability to archetype-based systems is in its infancy and interesting future work remains. Some staring points for future work is listed in the following paragraphs.

Bookmark usage in EHRs have only been scratched on the surface, future studies in real settings are needed to explore the implications and possibilities. Also, print-specific bookmarks could be created for identifying each EHR paper printout and included as QR (or other) code on the paper. It would then be easy to register the code with a camera and ask the system to retrieve later versions of the corresponding information if available.

If the bookmark URIs are to be short, opaque, and shared between users, it is not obvious how to shard bookmarks in an optimal way that also allows a user to efficiently list all their bookmarks, etc. Determining this will need further work.

A rudimentary HTTP-based test suite for testing implementations based on this design approach has been started, but it could get expanded to cover all the parts in a future openEHR Service specification for REST. It would automatically be applicable to all HTTP- and messaging-compliant implementations since those interfaces are not tied to any specific programming language.

Comparisons of log content and log granularity with healthcare laws and regulations will have to be made to see if it needs to be supplemented with more mechanisms to function as a complete auditable read-log. The audit-related RFC 3881, http://www.rfc3881.net/Resources/RFC3881.pdf, could be useful to consider in this context. Also, an investigation regarding if and how available HTTP server log analysis tools could be used for EHR access log analysis, should be done.

Shared contribution builds is another interesting potential future work. The current contribution builder design works best if a contribution build is personal and the user uses one device at a time for editing it. For more dynamic teamwork or multimodal or multidevice data entry, using systems based on Operational Transformation (OT) would likely enhance the user experience. OT is a lock-free resolution mechanism [33] that is used for example in collaborative systems like Google Docs and Apache Wave (formerly Google Wave). Since OT involves many small transactions a use of approaches like WebSockets for accessing shared contribution builds is anticipated. When explored properly, specific OT application recommendations should be added to the architecture.

The LiU EEE demonstrator implementation, detailed in Appendix A, is not intended for routine clinical use, but rather for prototyping and experimentation. At the time of publication, there is not yet sufficient material available to say that this architecture is useful for rapid prototyping by relevant target groups like students, software developers, and computer-savvy clinicians. The fact that we found it very efficient for creating the supplied demo pages does not constitute any unbiased proof, and further studies involving collaborative prototyping and desired target groups are needed.

## Conclusions

Using REST over HTTP addressed many architectural concerns regarding subdividing the implementation of the openEHR specifications into manageable pieces. This also gave additional benefits such as enabling rapid prototyping using regular web technologies and added a straightforward way of implementing bookmarking services to help clinicians share pointers into the EHR. Also, the HTTP server log can be used to monitor most user interactions with the system. An additional messaging component was needed to address event-based functionality, for example to notify decision support systems of about updates in an EHR. The proposed architecture is an openEHR service interface that can be used to implement and compose EHR systems using many different HTTP-capable platforms and programming languages.

Further work is needed to allow storage and efficient retrieval based on automated subsumption and post-coordinated terminology expressions. Since terminology binding adds complexity to the system, performance issues related to the interface between the EHR system and the terminology system will need further investigation.

Because of the ever increasing amount of patient data available, scaling is a critical aspect of EHR systems. REST was designed for large-scale, distributed systems, and in addition to that the sharding possibilities described in the paper can allow scaling out by adding more servers to share the work load.

The described architecture aims to address speed of access for end users, and enable creation of maintainable user interfaces. This paper indicates paths towards these goals, but does not by itself provide any proof thereof. Thus further experimental follow-up studies using the suggested approach are needed. This ‘software paper’ is not advocating the use of these practices in real healthcare IT systems until they have been further investigated and refined, instead the intention is to present the framework to a broader research community, welcoming further studies, critique and improvements!

## Availability and requirements

• Project name: LiU EEE

• Project home page and source code repository: https://github.com/LiU-IMT/EEE it may later get moved to a subproject somewhere under https://github.com/openEHR/

• Operating system(s): Platform independent

• Programming language: Java and JavaScript

• Other requirements: Java J2SE 5.0 or higher (and for some GUI examples an updated modern standards-compliant web browser like a recent version of Firefox, Safari, or Google Chrome)

• License: Apache 2 (permissive, commercially friendly open source)

• Any restrictions to use by non-academics: No

## **Appendix A: LiU EEE – Implementation details**

At Linköping University (LiU) we needed an Educational EHR Environment (EEE) for teaching and research. To facilitate cooperation with commercial and other actors, the implementation needed to be accessible as open source with a commercial-friendly, permissive license. By the time the design and development of LiU EEE started, we did not find alternatives that met our needs and decided to design the architecture described in the main manuscript and implement essential parts of it.

By the time of this publication the situation has changed for the better and several open source alternatives that explore different approaches to implement archetype-based EHR systems are available. Examples (programming language in parenthesis) are:

• EHRflex [34], http://ehrflex.sourceforge.net/ (Java)

• GastrOS [35], http://gastros.codeplex.com (.NET + C#)

• Open EHR-Gen, http://code.google.com/p/open-ehr-gen-framework/ (Groovy + Java)

• Opereffa, http://opereffa.com/ (Java)

Also, implementations in Ruby [36], http://openehr.jp/projects/show/ref-impl-ruby, and Python [37]https://launchpad.net/oshippy provide components for archetype-based EHR systems. The REST-based approach described in the main manuscript paper could potentially be used to compose EHR systems based on components from a mix of the above-mentioned projects.

### **Storage and representations**

LiU EEE stores EHR data as XML documents in an indexed XML database. This may not be the best long-time solution, especially not for population queries. An initial study [7] suggested that the tested XML database configurations without further optimizations were not suitable as persistence mechanisms for openEHR-based systems in production if population-wide ad hoc querying was needed. However, for individual focused clinical queries, corresponding to the ‘single record’ use case where patient ID was specified, the response times in the study were acceptable.

Using AQL [9,10] and an XML database provided a quick start for a proof of concept because openEHR XML schemas, serialization code, and example openEHR XML instances already existed. The XML-focused query language XQuery [26] was available as well. XQuery is path-based (as is AQL) and powerful enough to execute any query semantics AQL can express. An XML schema with additions needed for objects not covered by the official openEHR schema files is supplied in the LiU EEE source code. The current LiU EEE storage media type is XML, but clients can ask for other media types, and for example receive HTML and for some resources also JSON, YAML, or serialized Java objects. This is served by calling conversion mechanisms.

### **Frameworks used in the current LiU EEE implementation**

The LiU EEE implementation is mostly based on open source Java (server side) and JavaScript (client side) frameworks. Some significant currently used ones are listed below:

•The Restlet Lightweight REST framework, http://www.restlet.org/, that implements fundamental REST concepts as Java classes. Among other things it includes content and cache negotiation mechanisms. Interaction between Restlet components can run efficiently avoiding network calls by using ‘in process’ communication when deployed on the same computer, but can easily be configured to call components on other computers using HTTP when scaling out is needed.

•The openEHR Java Reference implementation [2] provides essential building blocks for openEHR-based EHR systems. It is used for managing archetypes and templates, as well as validation and conversion of EHR data.

•JavaCC, a Java Compiler Compiler, http://javacc.java.net/, was used for AQL parser generation

•Some XML databases tested [7] in conjunction with LiU EEE are: BaseX, http://basex.org/ and eXist-db, http://exist.sourceforge.net/

•FreeMarker, http://freemarker.sourceforge.net/, is used for generating (mostly HTML) pages by combining dynamic variable values with FreeMarker template files.

•JavaScript libraries used in the example user interface are primarily jQuery, http://jquery.com/, for data- and event-processing, and D3, http://d3js.org/, for information visualization.

### User interfaces

The version of LiU EEE published with this paper contains two kinds of user interfaces, one with example parts aimed for **end-users** such as clinicians and another one intended for **developers**.

Some of the provided example interfaces for end users of LiU EEE are partly shown in Figure 1 and Figure 4 in the main manuscript. They serve as suggestions and example GUI screens for client-side functionality exemplifying how to use the REST-based architecture. They do not expose the internals of the application to clinical end users; the Bookmarking section of the main manuscript explains some reasons why. The implemented LiU EEE summaries and graphical overview examples are based on AQL queries that are used to populate HTML pages with data and hyperlinks pointing to more detailed information.

**Figure 5:**
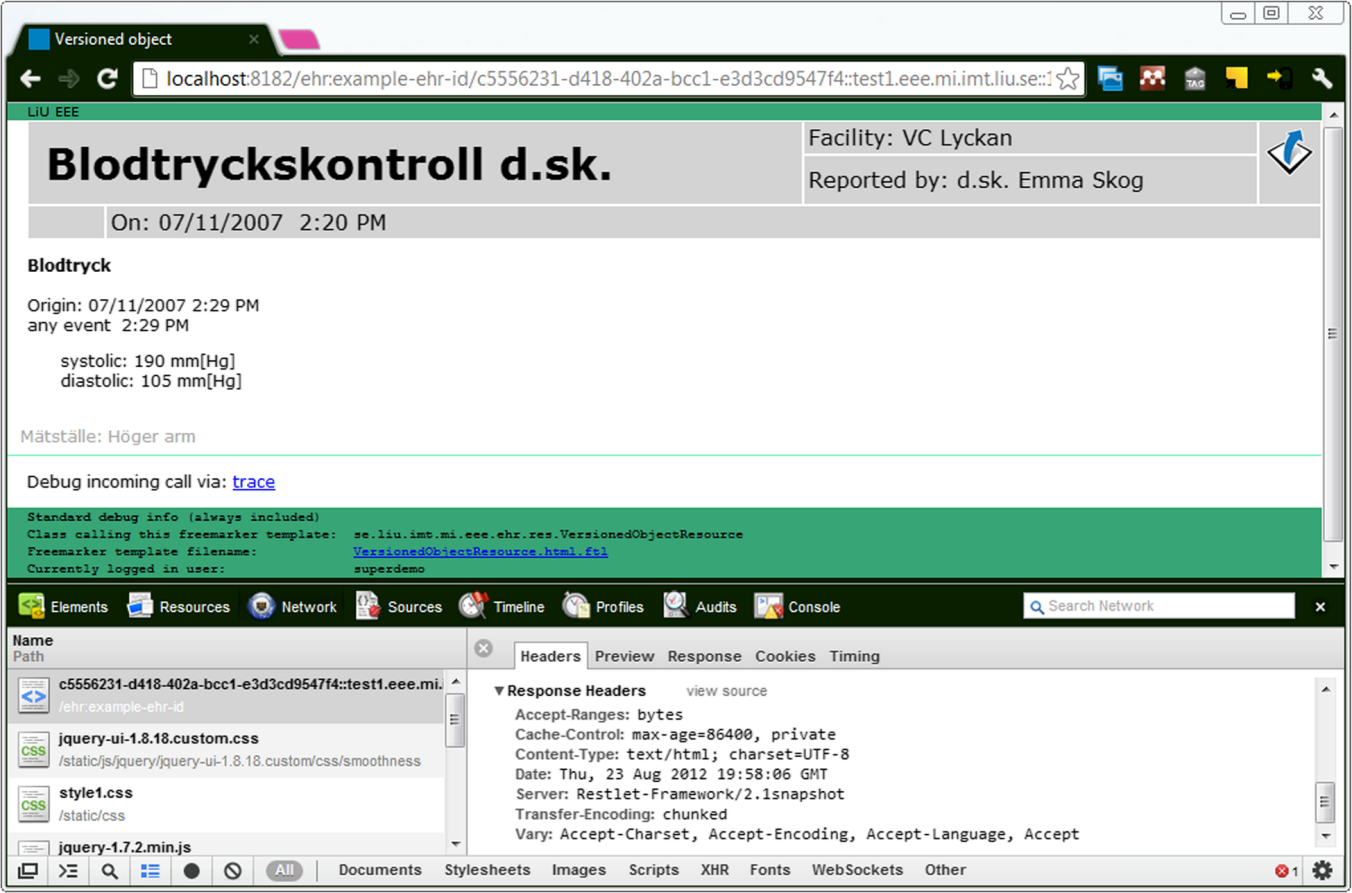
**Developer interface.** The top half of the image shows the interface for developers, here viewing a VERSION of a COMPOSITION and a footer with debug information that aids in understanding the implementation. The bottom half shows the network view in the debugging tool built into most WebKit-based browsers (here activated by right-clicking the page and selecting ‘Inspect Element’). Readers wanting to explore LiU EEE are encouraged to use these feature combinations.

The interfaces for developers, system technicians, and others wanting to learn what is ‘under the hood’, consist of simple web pages that expose the URIs and methods used to access data, often including forms, etc. Dynamically generated pages include a footer (similar to the one in Figure 5 with information about what Java classes and FreeMarker templates that were used in their creation. There are explaining pages available at most levels in the URI path hierarchy, so a call to /ehr/12344321/ (if there is such a patient) will return a page for developers. In most system deployments with real patient data, this developer interface should not be available or exposed to end users.

In addition to the interfaces there are also ‘Web Application Description Language’ (WADL) files that contain XML-formatted descriptions of the application. The formal WADL-formatted descriptions of URIs, methods, and representations are available to developers by calling URIs using the HTTP OPTIONS verb; these are also linked from the developer interface start page. The current WADL is automatically generated by the Restlet framework that introspects the Java code of LiU EEE. If a formal openEHR service specification targeted for REST is formulated, then the reverse process is likely: generating code stubs from openEHR specification-provided WADL in order to configure compatible systems (Figure 5).

### **AQL, query parsing, translation and hybrid queries**

The AQL grammar [38] was analyzed, transformed, and then converted and modified it to a file that could be consumed by JavaCC. The grammar file was then augmented with production rules generating XQuery corresponding to the specific storage format used in LiU EEE. (If other XML storage structures are used, these production rules need to be modified.)

Most of the graphical timeline examples provided in the LiU EEE implementation fetches ‘pure’ XML formatted responses and processes them in client side JavaScript code when generating views.

Another option available in LiU EEE are hybrid queries (as described in the “Implementation” section of the main manuscript) that combine AQL and XQuery. Some example hybrid queries supplied are:

•AQL embedded inside XQuery inside HTML, resulting in a HTML page being rendered as output directly from the database engine response.

•One example generates KML (a geography oriented XML dialect) projecting a graphical EHR overview in Google Earth using the principles outlined in a previous paper [39].

•Another query example produces RDF for the SMART platform, http://www.smartplatforms.org/, listing patient encounters [40]. Since all queries get stored and are assigned a SHA1-based URI in LiU EEE, these URIs can be used for routing, redirects etc. This means that a URI pattern like GET /records/{record_id}/encounters/ described in the SMART REST call specification [41] can, with very little coding effort, be routed to the encounter-fetching query. Similar translation-queries and URI mappings could be set up for other SMART API calls.

### Event driven messaging

The messaging semantics of messaging protocols like Simple Text Orientated Messaging Protocol (STOMP), http://stomp.github.com/, covers what is presently needed for event-based communication between LiU EEE components. A STOMP message contains plain text headers and may include a body in arbitrary format. STOMP clients are available for many programming languages. Components interacting with (or replacing) LiU EEE components can thus be written in any language environment that supports HTTP and STOMP.

### Limitations of initial open source implementation and future work

In order to reduce the time to sharing of findings and software, the LiU EEE implementation is limited in scope. Fine-grained security aspects such as access control lists have for example not been studied and implemented in detail. Import and export of EHR data to other openEHR systems using EHR extracts, etc., via network is not implemented either. Some other limitations and future work are discussed in the subsections below.

#### ***Performance***

Theoretical performance analysis can be of some help in system design, but is of limited value since the dependencies and mechanisms of implemented systems are often so complex that monitoring of real systems used in production is necessary to gain important insights [42]. At this stage, no EHR system based on our REST-based design for openEHR has been used in a realistic setting, so further studies are needed.

The current Java, HTML, and JavaScript implementations have not been optimized for performance. Performance profiling of the Java code and database query processing should also be done to find bottlenecks.

Implementations of MapReduce approaches to query and storage and high availability aspects are not reported in this paper, nor included in supplied code, but as a follow-up to the first storage performance study [7], experiments with open source distributed storage solutions based on Apache Hadoop’s, http://hadoop.apache.org/, HIVE project, Couch DB http://couchdb.apache.org/ and other solutions are currently being performed.

The JavaScript-parts of the interface would likely be helped by more organized development infrastructure and optimizing compilation provided by frameworks such as Closure, http://code.google.com/closure/. Graphics could also be optimized using CSS sprites, etc.

Regarding caching the LiU EEE implementation contains a rudimentary in-memory cache but can also be configured to use Memcache if such servers are available. Also worth noting regarding caching in the current LiU EEE implementation is that resources served under the subdirectory named ‘/static’ are automatically marked with the ‘Cache-Control: public’ HTTP header and a high ‘max-age’ to avoid repeated downloads.

#### ***Query translators***

The JavaCC format and tools proved somewhat cumbersome to work with. JavaCC was chosen primarily because it could be expected to be familiar to openEHR developers since it is already used for ADL parsing in the openEHR Java Reference implementation. Benefits and drawbacks of switching to another parser generator with better tooling support needs to be investigated.

#### ***Query return formats***

In addition to the XML-based query response formats currently implemented, it may be useful to develop for example JSON-based response formats once there are openEHR specifications for how to serialize openEHR object trees in those formalisms. Partial conversion routines for the XML query response results to JavaScript objects (via JSON) are provided in client side experimental GUI code.

#### ***Terminology bound querying***

In the development of LiU EEE, initial work [43] has been done on allowing storage and querying of coded data. In a demonstration of the abilities of the system, AQL queries allowed for querying of SNOMED CT coded data taking the subsumption hierarchy of the terminology into consideration.

For future development, a number of terminology related issues have to be considered. If the level of use of codes in patient information instances is high, the interface between the EHR system and any terminology server can be expected to be a bottleneck. Depending on the specific situation with regard to the ratio of number of relevant codes to the number of information instances, the optimal placement of the terminology querying function may vary. For example, querying a large set of documents for a single code is different from querying a limited set of documents for codes from a large hierarchy of codes. Also, the results of different terminology queries will have to be integrated with the query based on information structure, adding complexity to the querying task. Thus, testing different indexing techniques for coded information integrated with terminology servers needs to be explored.

#### ***Demographics and access control***

Demographic services and user account administration are rudimentary. The openEHR demographics and access control mechanisms have not been implemented but are needed to get a realistic functional system. How to best modify querying to respect (possibly fine-grained) access control lists is an interesting future work.

#### ***Embedded contribution builders***

It might be possible to make the Contribution Builder partly or fully embeddable in fairly ‘thin’ clients, thus improving performance and enabling temporary offline data entry. The Restlet framework is for example available both for Android and GWT (Google Web Toolkit).

## **Appendix B: Introduction to openEHR**

The openEHR project, described at http://www.openehr.org, has specified data structures and supporting semantics (clarifying the meaning) for the content of Electronic Health Records (EHRs). The formalisms in openEHR and the ISO 13606 standard are closely related. The EHR content is structured in a multi-level modeling approach including templates, archetypes, and a reference model (RM) [5] intended to improve semantic interoperability and reuse.

**Figure 6:**
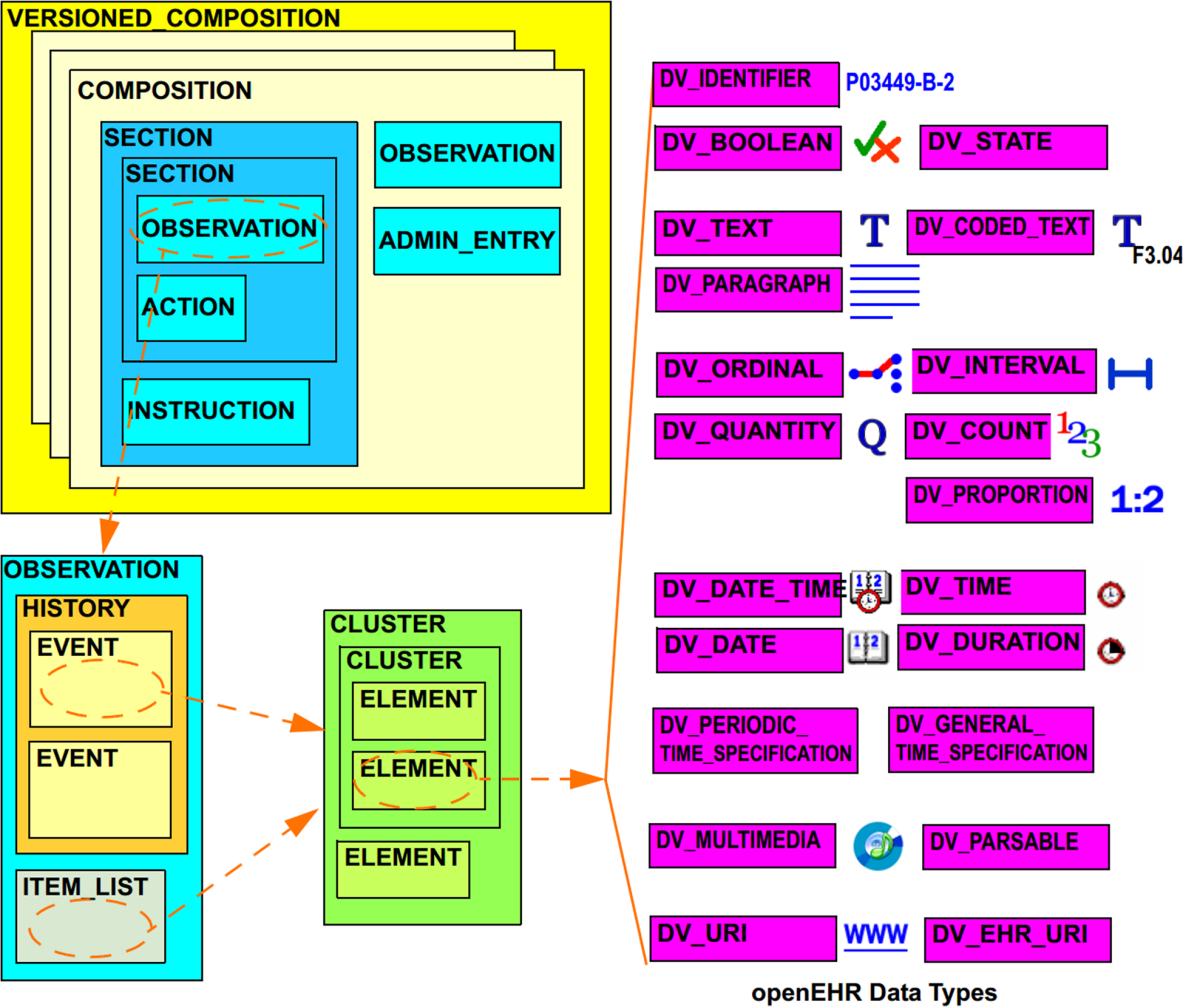
**openEHR reference model.** Reference model objects are building blocks that can be configured, constrained, and named by openEHR archetypes and templates forming document tree structures. *Image source: openEHR Architecture Overview (*[5]*page 28) used with permission.*

The idea of a layered modeling that separates technical infrastructure concerns from clinical concerns is not unique to the archetype approach – configurable template systems, for example as customizable forms, have been available in proprietary EHRs for a long time. However, they are seldom standardized and interoperable (Figure 6).

The technical **Reference Model (RM)** provides the foundational general building blocks (see Figure 6) that are then combined, named, and used in tree-like data structures according to rules and constraints defined in archetypes and templates. The RM aims to provide a common design for general data that is useful in many clinical settings, for example configurable data fields, units, time-points, user participations, and versioning [5].

Some openEHR RM structures are crucial in the suggested REST-based openEHR architecture, here capitalized as in the openEHR specifications:

• CONTRIBUTIONs can be thought of as a commit-log in a versioning system pointing to all objects updated in a single commit and containing metadata about who did what and when, together with an optional textual description.

• VERSIONED_OBJECTs are containers that keep track of VERSIONs of data objects like COMPOSITIONs (containing EHR data) and FOLDERs (optionally grouping COMPOSITIONS and other FOLDERs as a directory).

**Figure 7:**
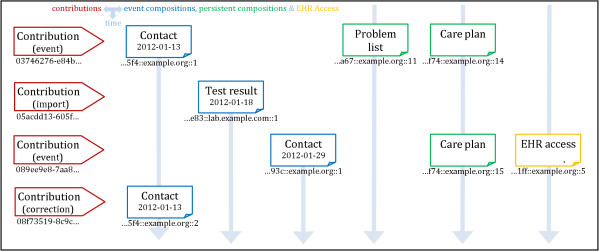
**EHR versioning.** Example EHR changes to VERSIONED_OBJECTs over time, committed and logged as contributions at four separate time points (the time axis goes downwards). COMPOSITIONs, FOLDER, EHR_ACCESS, and EHR_STATUS are all versioned using the same mechanism. Compositions can be flagged as being of type ‘event’ (blue above) intended for one-time recordings, or flagged as ‘persistent’ (green above) intended for long time continuously updated use. Abbreviated example identifiers are illustrated under the shapes. *The figure was inspired by Figure*8*in the openEHR architecture overview document*[5]*.*

These structures, as RM structures in general, have identifiers that can be used to access data. Some version changes are illustrated in Figure 7. The identifier of a versioned COMPOSITION is derived from its enclosing VERSION object.

An aspect that helps in replicated openEHR systems is that openEHR records are ‘append only’, so old VERSIONs are never physically changed or deleted. Logical deletion, if needed, is done inside the VERSIONED_OBJECT creating a new VERSION marked as deleted. The identifiers in the versioning system also include the ID of the EHR system creating a VERSION; this facilitates branching and merging of objects distributed over several care providers, etc. (Figure 7).

An **archetype** in openEHR and ISO 13606 contains metadata and a set of terms, rules, and constraints describing how to use the RM building blocks to create a clinically relevant data structure. One method often used in archetype design is trying to cover *all* possible aspects (maximal dataset) of a specific well-bounded clinical concept, such as the recording of blood glucose measurements or body weight (including details of measurement method, amount of clothing, etc.). The archetypes can then, for example when used for data entry, be combined with other archetypes to form larger composite structures [5].

An archetype can also contain language translations so that structured data entered using terms from the archetype in one language can automatically be displayed in another language.

A **template** (in the openEHR sense of the word) is used to combine several archetypes into a larger structure, intended for a specific or local use case, to be used as the basis for a clinical system entry form in a certain EHR system, for example an in-patient admission form. A template can also further constrain, remove, or set default values from the archetypes and the reference model it builds upon. Templates cannot remove mandatory fields or ‘add’ new fields, just use and further constrain concepts defined by existing archetypes. Thus all EHR data based on a template must also be valid instances in accordance with the archetypes included in the template [5].

The intentional split between archetypes and templates is primarily for practical and pedagogical reasons since they have different purposes. Due to their maximal dataset nature, archetypes are supposed to be reusable and created regionally, nationally, or internationally when possible [44].

**Figure 8:**
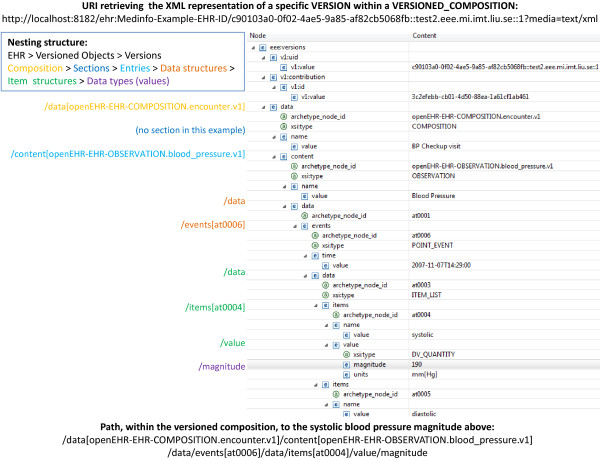
**Document tree and paths.** A simplified document tree rendered from an XML representation of a VERSIONED_COMPOSITION. An example path is illustrated using the same color encoding as the RM building blocks in Figure 6. Paths and values can be used in queries to extract and display data in the EHR system.

The openEHR RM, like any labeled tree data structure, can be traversed using **paths.** Every part (node) of an archetype-based data structure in an EHR is addressable and thus retrievable by a path containing a concatenation of traversed RM attribute names and within brackets archetype IDs and subsequent node-IDs. Templates don’t add data with any other paths than the ones available in the archetypes. Thus data originating from systems using different templates but the same archetype can be retrieved using the same query. Figure 8 exemplifies paths in a data structure (Figure 8).

**Figure 9:**
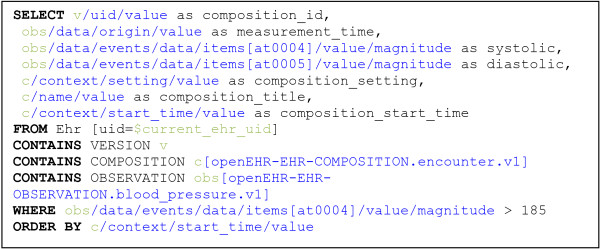
**AQL query example.** Example of a query in ‘archetype query language’ (AQL).

One of the domain specific languages (DSLs) designed for archetype-based queries is the ‘archetype query language’ (AQL) [9,10] that uses an XPath-inspired syntax exemplified in Figure 9.

The AQL example in Figure 9 selects and labels some specific return measurement values and metadata from all blood pressures with a systolic value above 185 that were recorded as part of patient encounters in a specific EHR. Variables are in green text (the value of the variable $current_ehr_uid is supplied by the calling program context and contains the EHR ID of a specific patient), paths are blue and AQL commands **BOLD**.

## Abbreviations

AM: Archetype model; EHR: Electronic health record; HTML: Hypertext markup language; JSON: Javascript object notation; PHR: Personal health record; QR Code: Quick response code, a type of matrix barcode; REST: Representational state transfer; RM: Reference model; STOMP: Simple text orientated messaging protocol; XML: Extensible markup language.

## Competing interests

The authors declare that they have no competing interests.

## Authors' contributions

ES Did the initial drafting of major parts of the manuscript and was the coordinating editor. ES developed the idea of applying REST to openEHR and designed and implemented major parts of LiU EEE in cooperation primarily with ME, MN, and DK. RC extended the openEHR Java reference implementation to include archetype- and template-based validation as well as the RM-instance/skeleton builder components. RC also drafted the manuscript parts about these components. MN and ME designed and implemented the AQL parser and the AQL to XQuery translator. MN, ME, and ES designed the initial bookmarking features. MN pointed out the need for privacy preserving opaque bookmark URIs. Detailed design and implementation was then finished by ES and ME. HÖ supervised parts of the design and development and contributed to major clarifications of the manuscript All authors have commented, contributed to, critically reviewed, and approved the manuscript. Parts of the text describing the bookmarking features have been previously published as a poster abstract at MIE 2011 in Oslo: *Bookmarking Service Considerations for an Archetype-Based EHR Using REST,* by ES, MN, ME, DK, and HÖ.

## Pre-publication history

The pre-publication history for this paper can be accessed here:

http://www.biomedcentral.com/1472-6947/13/57/prepub
